# Predictors of early morbidity and mortality in newly diagnosed multiple myeloma: data from five randomized, controlled, phase III trials in 3700 patients

**DOI:** 10.1038/s41375-023-02105-6

**Published:** 2023-12-07

**Authors:** Elias K. Mai, Thomas Hielscher, Uta Bertsch, Hans J. Salwender, Sonja Zweegman, Marc S. Raab, Markus Munder, Lucia Pantani, Katia Mancuso, Peter Brossart, Meral Beksac, Igor W. Blau, Jan Dürig, Britta Besemer, Roland Fenk, Peter Reimer, Bronno van der Holt, Mathias Hänel, Ivana von Metzler, Ullrich Graeven, Carsten Müller-Tidow, Mario Boccadoro, Christof Scheid, Meletios A. Dimopoulos, Jens Hillengass, Katja C. Weisel, Michele Cavo, Pieter Sonneveld, Hartmut Goldschmidt

**Affiliations:** 1 Department of Internal Medicine V, University Hospital Heidelberg, Heidelberg, Germany; 2 Division of Biostatistics, German Cancer Research Center (DKFZ), Heidelberg, Germany; 3 National Center for Tumor Diseases (NCT) Heidelberg, Heidelberg, Germany; 4 Tumorzentrum Asklepios Hamburg, AK Altona and AK St. Georg, Hamburg, Germany; 5 Department of Hematology, Amsterdam UMC, Vrije Universiteit Amsterdam, Cancer Center Amsterdam, Amsterdam, Netherlands; 6 Department of Internal Medicine III, University Medical Center Mainz, Mainz, Germany; 7 IRCCS Azienda Ospedaliero-Universitaria di Bologna, Istituto di Ematologia “Seràgnoli”, Dipartimento di Medicina Specialistica, Diagnostica e Sperimentale, Azienda Ospedaliero-Universitaria di Bologna, Università di Bologna, Bologna, Italy; 8 Department of Internal Medicine III, University Hospital Bonn, Bonn, Germany; 9 Department of Hematology, Ankara University School of Medicine, Ankara, Turkey; 10 Medical Clinic, Charité University Medicine Berlin, Berlin, Germany; 11 Department of Hematology, University Clinic Essen, Essen, Germany; 12 Department of Hematology, Oncology and Immunology, University Hospital Tübingen, Tübingen, Germany; 13 Department of Hematology, Oncology and Clinical Immunology, University Hospital Düsseldorf, Düsseldorf, Germany; 14 Klinik für Hämatologie, Evangelische Kliniken Essen Mitte, Evangelisches Krankenhaus Essen-Werden, Essen, Germany; 15 HOVON Data Center, Department of Hematology, Erasmus MC Cancer Institute, Rotterdam, Netherlands; 16 Department of Internal Medicine III, Klinikum Chemnitz, Chemnitz, Germany; 17 Medical Clinic II, University Hospital Frankfurt am Main, Frankfurt am Main, Germany; 18 Department of Internal Medicine I, Hospital Maria Hilf GmbH, Mönchengladbach, Germany; 19 Myeloma Unit, Division of Hematology, University of Torino, Azienda Ospedaliero-Universitaria Città della Salute e della Scienza di Torino, Turin, Italy; 20 Department of Internal Medicine I, University Hospital Cologne, Cologne, Germany; 21 Department of Clinical Therapeutics, School of Medicine, National and Kapodistrian University of Athens, Athens, Greece; 22 Roswell Park Comprehensive Cancer Center, Buffalo, NY USA; 23 Department of Oncology, Hematology and Bone Marrow Transplantation with Section of Pneumology, University Medical Center Hamburg-Eppendorf, Hamburg, Germany; 24 Department of Hematology, Erasmus MC Cancer Institute, Rotterdam, Netherlands

**Keywords:** Myeloma, Risk factors

## Abstract

Early morbidity and mortality affect patient outcomes in multiple myeloma. Thus, we dissected the incidence and causes of morbidity/mortality during induction therapy (IT) for newly diagnosed multiple myeloma (NDMM), and developed/validated a predictive risk score. We evaluated 3700 transplant-eligible NDMM patients treated in 2005–2020 with novel agent-based triplet/quadruplet IT. Primary endpoints were severe infections, death, or a combination of both. Patients were divided in a training (*n* = 1333) and three validation cohorts (*n* = 2367). During IT, 11.8%, 1.8%, and 12.5% of patients in the training cohort experienced severe infections, death, or both, respectively. Four major, baseline risk factors for severe infection/death were identified: low platelet count (<150/nL), ISS III, higher WHO performance status (>1), and age (>60 years). A risk score (1 risk factor=1 point) stratified patients in low (39.5%; 0 points), intermediate (41.9%; 1 point), and high (18.6%; ≥2 points) risk. The risk for severe infection/death increased from 7.7% vs. 11.5% vs. 23.3% in the low- vs. intermediate- vs. high-risk groups (*p* < 0.001). The risk score was independently validated in three trials incorporating quadruplet IT with an anti-CD38 antibody. Our analyses established a robust and easy-to-use score to identify NDMM patients at risk of severe infection/death, covering the latest quadruplet induction therapies. Trial registrations: HOVON-65/GMMG-HD4: EudraCT No. 2004-000944-26. GMMG-MM5: EudraCT No. 2010-019173-16. GMMG-HD6: NCT02495922. EMN02/HOVON-95: NCT01208766. GMMG-HD7: NCT03617731.

## Introduction

Clinical outcomes markedly improved over the past two decades in multiple myeloma (MM) [1, 2]. Yet, the risk of early morbidity and mortality can limit the therapy-related benefit of long-term disease control in a substantial number of patients. Various studies have demonstrated that the risk for morbidity and mortality from adverse events, mainly severe infections, during treatment initiation exceeds the risk from MM progression [3–7].

Risk for severe infections in MM is caused by secondary immunodeficiency [3, 4] and potentiated by novel treatments, including monoclonal antibodies (mAb; i.e. anti-CD38 [daratumumab, isatuximab] or anti-SLAMF7 [elotuzumab]), immunomodulatory agents (IMiDs; i.e. thalidomide, lenalidomide), proteasome inhibitors (PI; i.e. bortezomib, carfilzomib), and accompanying steroids [8–10]. Host factors such as the patient´s performance status, frailty, and comorbidities further aggravate the risk of early infections, treatment discontinuation, and death [11, 12].

Thus, better characterization and improved prediction of individual risk for severe infections and death are essential to develop advanced preventive measures. To date, no large analyses have evaluated early morbidity and mortality in transplant-eligible patients with newly diagnosed MM (NDMM) in the era of modern myeloma treatment.

The present multi-cohort analysis included 3700 transplant-eligible patients with NDMM from five multi-center, phase III trials. All patients received novel agent-based triplet or quadruplet induction therapies. The aims of this study were (i) to dissect the incidence, timing, and causes of morbidity and mortality during induction therapy, and (ii) to develop and validate a predictive risk score to identify patients at excessive risk of severe infections and death during the early treatment phase.

## Patients and Methods

### Study cohorts

Trials included in this study were split into a training and validation cohort. The training cohort included three randomized, multi-center, phase III trials from the German-speaking Myeloma Multicenter Group (GMMG-HD4 [13, 14], EudraCT No. 2004-000944-26, GMMG-MM5 [15, 16] EudraCT No. 2010-019173-16, and GMMG-HD6 [17, 18] NCT02495922). The validation cohort for the proposed risk score comprised the Dutch–Belgian Cooperative Trial Group for Hematology Oncology (HOVON) HO65 [13, 14] (EudraCT No. 2004-000944-26), the European Myeloma Network (EMN) 02/HO95 [19] (NCT01208766), and the GMMG-HD7 [20] (NCT03617731) trials.

All patients included in this analysis had untreated NDMM and were considered eligible for induction therapy followed by high-dose melphalan (200 mg/m^2^) and autologous stem cell transplantation. Patients received at least a bortezomib-containing triplet induction regimen: bortezomib-doxorubicine-dexamethasone (PAD; HD4, MM5, HO65) or bortezomib-cyclophosphamide-dexamethasone (VCD; MM5, EMN02/HO95). In the HD6 and HD7 trials, induction therapy included lenalidomide-bortezomib-dexamethasone (RVd) with or without the anti-SLAMF7 mAb elotuzumab (HD6) or anti-CD38 mAb isatuximab (HD7). Information on trials, induction therapy schedules, number of cycles, and recommended use of antibacterial prophylaxis is summarized in Supplementary Table S1. All analyses were performed on individual patient-level data. Trials were conducted according to the European Clinical Trial Directive and the Declaration of Helsinki, and were approved by the local ethics committees. All patients gave written informed consent for participation in the respective clinical trials.

### Definitions, assessments, and objectives

Patients who received at least one dose of trial medication were included and analyzed as treated. Eight patients from the HD6 trial receiving > 4 induction therapy cycles were excluded from the analysis. The induction period was defined from the first until the last dose of induction treatment plus 30 days, or until the start of stem-cell mobilization.

The primary endpoints of the study were rates of severe infection, death from any cause, or a combined endpoint of severe infection/death from any cause, whichever occurred first, during the induction period. Severe infections were defined as any infection of grade ≥3 according to the National Cancer Institute Common Terminology Criteria for Adverse Events (CTCAE). In case of multiple severe infections, the first one was counted.

The following variables were analyzed for their effect on endpoints: patient age (≤60 vs. >60 years), sex (male vs. female), World Health Organization (WHO) performance status (0–1 vs*.* >1), body mass index (BMI; ≤30 vs. >30 kg/m^2^), white blood cell count (≥4.0 vs. < 4.0/nL), hemoglobin value (≥10.0 vs. <10.0 g/dL), platelet count (≥150 vs. <150/nL), serum creatinine (≤2.0 vs. >2.0 mg/dL), calcium (≤2.75 vs. >2.75 mmol/L), C-reactive protein (≤5.0 vs. >5.0 mg/L), lactate dehydrogenase (LDH; normal vs. >upper limit of normal [ULN]), International Staging System (ISS; stages I/II vs. III), cytogenetics by fluorescence in-situ hybridization (standard vs. high risk; defined as at least one of the following [cut-off ≥10% of cells]: del17p or t(4;14), or amp(1q21) [>3 copies]), severe infections of CTCAE grade ≥3 during induction therapy (no vs. yes), and severe thromboembolic events of CTCAE grade ≥ 3 during induction therapy (no vs. yes).

### Statistics and general methods

Fisher’s exact test and Wilcoxon test were used to compare categorical and continuous variables between trials. Univariable and multivariable logistic regression models were used to assess the impact of risk factors on endpoints, depicted as odds ratio (OR) and 95% confidence interval (95% CI). In case of complete separation, logistic regression with Firth correction was applied. In all pooled analyses, the trial effect was included in the model. Likelihood-ratio test between model, with and without parameter-trial interaction term, was used to assess heterogeneity of effect between trials. For multivariable models, multiple imputations of missing values (100 bootstrap samples) for baseline variables were done by applying the multivariate imputations using the chained equations (mice) algorithm [21]. *P* values from the univariable analysis were adjusted for multiple testing using Benjamini-Hochberg correction to control the false discovery rate. *P* values < 0.05 were considered statistically significant. Analyses were performed with the statistical software R 4.0 (R Foundation for Statistical Computing, Vienna, Austria; https://www.R-project.org/).

## Results

### Patient characteristics and treatment completion

The training cohort included 1333 patients (192, 596, and 545 patients from the HD4, MM5, and HD6 trials, respectively). The median patient age was 58 (range 27–70) years with 534 (40.1%) patients aged > 60 years. The median duration of induction treatment was 89 (range 2–281) days. In total, 1261 (94.6%) patients completed regular induction treatment and 1103 (91.9%) received antibacterial prophylaxis. The baseline characteristics of patients in the training cohort are listed in Supplementary Table S2.

### Incidence, timing, and localization of severe infections and death during induction therapy

**Fig. 1:**
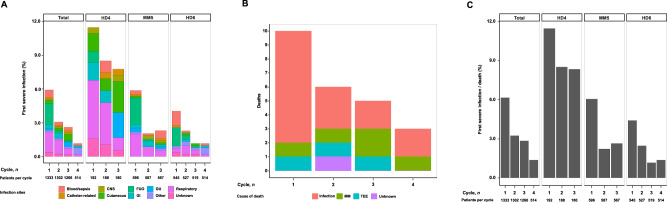
Incidence of severe infections and deaths or a combination of both during induction therapy in the training cohort. **A** Frequency of the first severe infection per induction cycle in the overall training cohort and every single trial (HD4, MM5, HD6). The colors indicate the primary infection sites. **B** Incidence of death per induction cycle in the overall training cohort. The colors indicate the leading causes of death. **C** Incidence of severe infections and death per induction cycle in the overall training cohort and each single trial (HD4, MM5, and HD6). CNS central nervous system, FUO fever of unknown origin, GI gastrointestinal, GU genitourinary, MM multiple myeloma, TEE thromboembolic event.

In the training cohort, 158/1333 (11.8%) patients had severe infections, 24/1333 (1.8%) patients died, and 167/1333 (12.5%) patients experienced a severe infection and/or death (Supplementary Table S3). Incidence of severe infections decreased in subsequent trials (HD4: 52/192 [27.1%], MM5: 60/596 [10.1%], and HD6: 46/545 [8.4%] patients; Fig. 1A). Overall and in every single trial, infections were the most common cause of death (15/24 [62.5%] deaths; Supplementary Table S3, Fig. 1B).

The majority of severe infections, deaths, or a combination of both occurred during the first two induction cycles: 119/158 (75.3%), 16/24 (66.7%), and 124/167 (74.3%; Fig. 1A–C). The median time from the start of induction therapy to the first severe infection, death, or a combination of both was short (severe infections: 36 [range 1–119] days; death: 66 [range 14–169] days; severe infection/death: 37 [range 1–146] days).

Common sites of infections were respiratory (48/158 [30.4%] patients), fever of unknown origin (29/158 [18.4%] patients), bloodstream/sepsis (18/158 [11.4%] patients), cutaneous (14/158 [8.9%] patients), and gastro-intestinal (13/158 [8.2%] patients; Fig. 1A).

### Identification of factors influencing the risk of severe infections, death, or the combined endpoint of severe infection/death during induction therapy

**Fig. 2:**
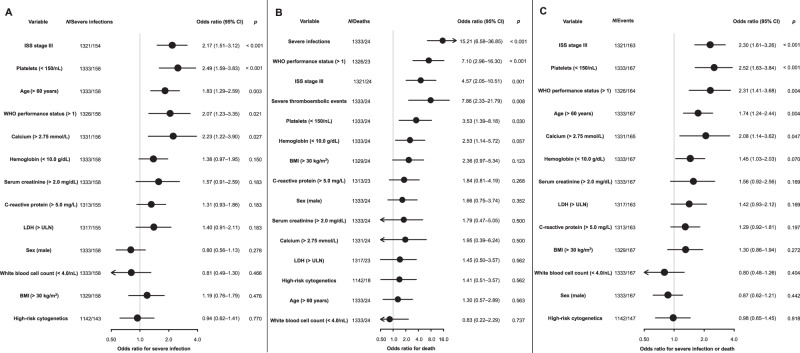
Logistic regression analyses on factors influencing the risk of severe infections, death or the combined endpoint of severe infections/deaths during induction therapy in the training cohort. Forest plots on factors influencing **A** risk of severe infections, **B** risk of death, and **C** risk of severe infections and/or death. All logistic regression analyses accounted for trial effects. *P* values from the univariable analysis were adjusted for multiple testing. BMI body mass index, ISS International Staging System, LDH lactate dehydrogenase, ULN upper limit of normal, WHO World Health Organization.

We aimed to identify risk factors associated with all three endpoints. Owing to the relatively small number of events, analyses were conducted in the pooled training cohort, accounting for trial effects (Fig. 2). The effects of risk factors for each trial included in the training cohort (HD4, MM5, HD6) are shown in Supplementary Fig. S1.

Low platelet count (OR = 2.49, 95% CI: 1.59–3.83, *p* < 0.001), elevated serum calcium (OR = 2.23, 95% CI: 1.22–3.90, *p* = 0.027), ISS stage III (OR = 2.17, 95% CI: 1.51–3.12, *p* < 0.001), WHO performance status >1 (OR = 2.07, 95% CI: 1.23–3.35, *p* = 0.021), and age >60 years (OR = 1.83, 95% CI: 1.29–2.59, *p* = 0.003; Fig. 2A) were major risk factors for severe infections during induction therapy.

Factors with the strongest association with risk of death were grade ≥ 3 adverse events during induction therapy: severe infections (OR = 15.21, 95% CI: 6.58–36.85, *p* < 0.001) and thromboembolic events (OR = 7.86, 95% CI: 2.33–21.79, *p* = 0.008). Baseline parameters closely associated with increased risk of death during induction therapy were WHO performance status > 1 (OR = 7.10, 95% CI: 2.96–16.30, *p* < 0.001), ISS stage III (OR = 4.57, 95% CI: 2.05–10.51, *p* = 0.001), and low platelet count (OR = 3.53, 95% CI: 1.39–8.18, *p* = 0.030, Fig. 2B).

Risk factors for the combined endpoint of severe infection/death were shared with the two separate endpoints: low platelet count (OR = 2.52, 95% CI: 1.63–3.84, *p* < 0.001), WHO performance status > 1 (OR = 2.31, 95% CI: 1.41–3.68, *p* = 0.004), ISS stage III (OR = 2.30, 95% CI: 1.61–3.26, *p* < 0.001), elevated serum calcium (OR = 2.08, 95% CI: 1.14–3.62, *p* = 0.047), and age >60 years (OR = 1.74, 95% CI: 1.24–2.44, *p* = 0.004, Fig. 2C).

### Multivariable model on predictors for risk of severe infection/death during induction therapy

Based on the findings from the logistic regression analyses, we aimed to confirm significant risk factors for the most clinically relevant, combined endpoint, severe infection/death, in a multivariable model. To account for parameters commonly used in clinical practice to assess infection, low baseline white blood cell counts and elevated C-reactive protein levels were included in the analysis as well. The multi-variable model identified four independent risk factors for the combined endpoint of severe infection/death during induction therapy: low platelet count (OR = 2.05, 95% CI: 1.28–3.33, *p* = 0.003), ISS stage III (OR = 1.93, 95% CI: 1.23–2.96, *p* = 0.004), WHO performance status > 1 (OR = 1.83, 95% CI: 1.10–3.08, *p* = 0.021), and age > 60 years (OR = 1.73, 95% CI: 1.22–2.43, *p* = 0.002; Table 1).

**Table 1:** Multivariable model on factors influencing the combined endpoint of severe infection/death during induction therapy in the training cohort.

Variable	Odds ratio	95% Confidence interval	*p*
Platelets (<150/nL)	2.05	1.28–3.33	0.003
ISS (stage III)	1.93	1.23–2.96	0.004
WHO performance status (>1)	1.83	1.10–3.08	0.021
Age (>60 years)	1.73	1.22–2.43	0.002
Calcium (>2.75 mmol/L)	1.59	0.82–2.84	0.174
BMI (>30.0 kg/m^2^)	1.29	0.83–1.93	0.258
Hemoglobin (<10.0 g/dl)	1.02	0.68–1.51	0.935
C-reactive protein (>5.0 mg/dL)	1.01	0.70–1.45	0.948
LDH (>ULN)	0.98	0.59–1.44	0.756
Sex (female)	0.81	0.57–1.15	0.238
White blood cell count (<4.0/nL)	0.78	0.46–1.26	0.324
Serum creatinine (>2.0 mg/dL)	0.70	0.38–1.27	0.249
GMMG-MM5	0.26	0.17–0.40	<0.001
GMMG-HD6	0.23	0.15–0.37	<0.001

*BMI* body mass index, *ISS* International Staging System, *LDH* lactate dehydrogenase, *GMMG* German-speaking Myeloma Multicenter Group, *ULN* upper limit of normal, *WHO* World Health Organization.

### A novel risk score to predict severe infection/death during novel agent-based induction therapy in transplant-eligible NDMM patients

**Fig. 3:**
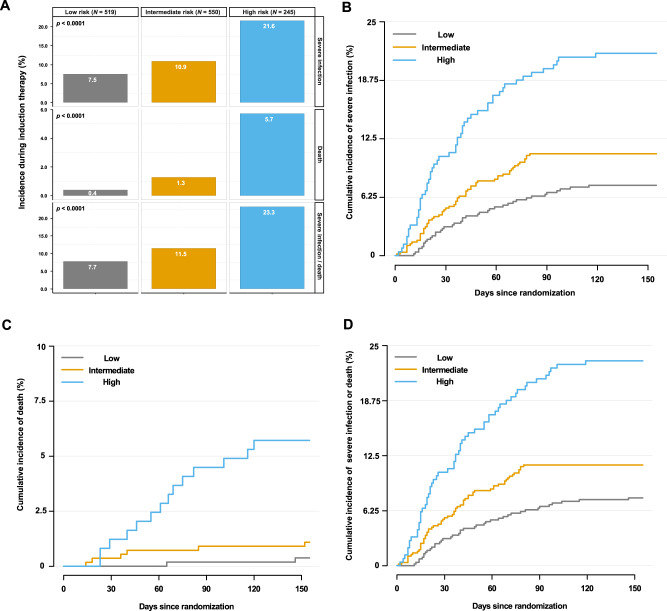
A novel risk score to predict severe infections/death during novel agent-based induction therapy in transplant-eligible NDMM patients. **A** Bar plots on incidence of severe infections, death, or both during induction therapy within the low-, intermediate- and high-risk groups in the training cohort. **B** Incidence of severe infections during induction therapy within the low-, intermediate- and high-risk groups. **C** Incidence of death during induction therapy within the low-, intermediate- and high-risk groups. **D** Incidence of severe infections/death during induction therapy within the low-, intermediate-, and high-risk groups. NDMM newly diagnosed multiple myeloma.

A sum score was built based on the identified four major risk factors. Each risk factor (low platelet count < 150/nL, ISS stage III, WHO > 1, and age > 60 years) was counted as 1 point. Three groups were identified by additive scoring: low risk (0 points), intermediate risk (1 point), and high risk (≥2 points). Risk groups included 519/1314 (39.5%), 550/1314 (41.9%), and 245/1314 (18.6%) patients in the low-, intermediate- and high-risk group, respectively (Fig. 3A).

The risk score predicted increasing risk for severe infection (low risk 7.5% vs. intermediate risk 10.9% vs. high risk 21.6%, *p* < 0.0001), death (low risk 0.4% vs. intermediate risk 1.3% vs. high risk 5.7%, *p* < 0.0001), and the combined endpoint of severe infection/death (low risk 7.7% vs. intermediate risk 11.5% vs. high risk 23.3%, *p* < 0.0001) during induction therapy (Fig. 3A). Cumulative incidences of the three endpoints according to risk groups are shown in Fig. 3B–D. Severe infection/death rates during the induction period doubled from the intermediate- to high-risk group (11.5% vs. 23.3%, OR = 2.34, 95% CI: 1.46–3.76, *p* < 0.001) and tripled from the low- to high-risk group (7.7% vs. 23.3%, OR = 3.63, 95% CI: 2.15–6.13, *p* < 0.001; Fig. 3B, Supplementary Table S4).

### Validation of the novel risk score to predict severe infection/death during novel agent-based induction therapy in transplant-eligible NDMM patients

In total, 2367 patients were included in the validation cohort (HO65: 218; EMN02/HO95:1491; HD7: 658). Median patient age was 57, 58, and 59 years (ranges 31–65, 28–66, 26–70 years) in the HO65, EMN02/HO95, and HD7 trial, respectively. The risk factor distributions included in the risk score are listed in Supplementary Table S5.

Severe infection/death during induction therapy occurred in 61/218 (28.0%), 118/1491 (7.9%), and 81/658 (12.3%) patients in the HO65, EMN02/HO95, and HD7 trials, respectively (Supplementary Table S6). Ninety/193 (46.6%), 597/1471 (40.6%), and 260/656 (39.6%) patients were in the low-risk group vs. 65/193 (33.7%), 556/1471 (37.8%), and 264/656 (40.2%) patients in the intermediate-risk group vs. 38/193 (19.7%), 318/1471 (21.6%), and 132/656 (20.1%) of evaluable patients in the high-risk group of the HO65, EMN02/HO95, and HD7 trials, respectively.

**Fig. 4:**
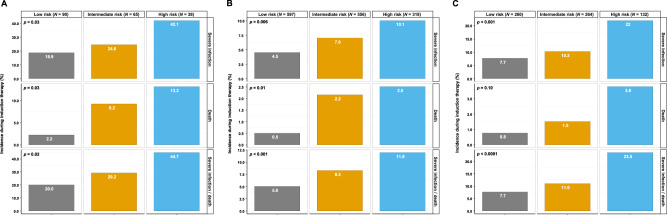
Validation of the risk score to predict severe infections and/or death during induction therapy in transplant-eligible NDMM patients in the HO65, EMN02/HO95, and HD7 trials. Validation of the risk score in the **A** HO65 trial, **B** EMN02/HO95 trial, and **C** HD7 trial. Bar plots show the incidence of severe infections, death, or both during induction therapy according to low-, intermediate-, and high-risk groups. NDMM newly diagnosed multiple myeloma.

The risk score was highly predictive, in all three cohorts independently, for the combined endpoint of severe infection/death (HO65: *p* = 0.02; EMN02/HO95: *p* < 0.001; HD7: *p* < 0.001; Fig. 4A–C). Patients’ risk for severe infection/death during induction therapy in the HO65 and EMN02/HO95 trials more than doubled in the low-risk vs. high-risk group (20.0% vs. 44.7%, OR = 3.24, 95% CI: 1.21–8.65, *p* = 0.005; Fig. 4A, Supplementary Table S7 and 5.0% vs. 11.9%, OR = 2.56, 95% CI: 1.41–4.66, *p* < 0.001; Fig. 4B, Supplementary Table S8, respectively) and tripled in the HD7 trial (7.7% vs. 23.5%, OR = 3.68, 95% CI 1.78–7.62, *p* < 0.001; Fig. 4C, Supplementary Table S9). For completeness, results for the other endpoints (severe infection, death) are shown in Supplementary Tables S7–S9.

## Discussion

Our results demonstrate that a combination of parameters readily accessible in the clinic–low platelet count (<150/nL), ISS stage III, WHO > 1, and age >60 years–consistently predicted risk of severe infection and severe infection/death in patients treated with concurrent, modern induction therapies, including quadruplets with an anti-CD38 mAb. To our knowledge, this is the largest, pooled analysis of individual patient-level data on early morbidity and mortality during novel agent-based induction therapy in transplant-eligible patients with NDMM, comprising 3700 patients treated between 2005 and 2020. Induction therapies in our dataset included a broad variety of widely used standard-of-care [22] triplet combinations such as RVd and VCD, as well as quadruplet combinations with the anti-CD38 mAb isatuximab plus RVd. Thus, the validated risk score can be considered a novel important tool to inform clinicians on the individual risk of early morbidity and mortality in patients with NDMM. Furthermore, it can serve as a benchmark in the design of future prospective clinical trials aiming to reduce early morbidity/mortality or tailor supportive care.

In line with prior analyses [5–7, 23, 24], our study showed that severe infections remain the major cause of early morbidity and mortality in NDMM, and by far exceed the risk of disease progression. This holds true, even though the incidence of severe infections and death has decreased over time in subsequent trials with the introduction of novel agents and the use of antibacterial prophylaxis in most patients (i.e., 91.9% in our training cohort). Thus, preventing early severe infections and death is paramount to achieving optimal outcomes in MM patients.

The composition of our risk score highlights that both disease-specific factors and tumor burden (ISS stage III, low platelet count), as well as host factors (poor performance status, older age), contribute to early morbidity and mortality. A recent study including 1347 pooled NDMM patients (of whom 847 were transplant-eligible) from the Spanish study group found similar predictors for severe infections (serum albumin, MM immunoglobulin subtype, male sex, European Cooperative Oncology Group [ECOG] performance status) [23]. The study evaluated mostly triplet induction therapies, including an IMiD and a PI, but no anti-CD38 mAbs. In this analysis, 11.4% of patients experienced a severe infection within 4 months from treatment initiation [23] compared to 11.8% during induction therapy in our training cohort. The varying risk factors identified in the Spanish study, as compared to our analysis, likely reflect differences between the cohorts investigated, such as patients´ characteristics and eligibility for autologous stem cell transplantation. Another score to predict early, severe infections in transplant-ineligible patients with NDMM derived from the FIRST trial (NCT00689936, 1613 patients), which included IMiD-only based therapies (without mAbs), mostly doublets [6]. Severe infections occurred in 11.9% of these patients during the first 4 months of treatment. Factors predicting the risk of infection in this study were ECOG performance status, beta-2 microglobulin, LDH, and hemoglobin values. Compared with these two earlier studies, our study is the only one including up-to-date, novel agent-based therapies with quadruplet regimens incorporating mAbs, IMiDs, and PIs and such a large number of patients.

Our study has a few limitations. It includes rather young and fit transplant-eligible NDMM patients treated within clinical trials. However, such systematically evaluated and detailed safety and endpoint data are rarely available outside of clinical trials.

Further, we could not evaluate the impact of antibacterial prophylaxis, since the majority of patients received antibacterial prophylaxis during induction therapy. Despite the positive results on the use of antibacterial prophylaxis with levofloxacin in the TEAMM trial (ISRCTN51731976) [25], its broad use in clinical routine remains controversial. Foremost, it remains an open question whether the use of antibacterial prophylaxis is only beneficial in patients at high risk of severe infections. For example, the TEAMM trial did not provide evidence of a clear benefit with levofloxacin use vs. placebo in younger, transplant-eligible patients or patients receiving cotrimoxazole prophylaxis [25]. Our risk score would allow stratification of patients in clinical trials investigating strategies to prevent infections, including antibacterial prophylaxis. Further, the optimal duration of antibacterial prophylaxis is not known and possible toxic effects (i.e. levofloxacin-induced tendinopathy and neuropathy) should be considered, especially in MM patients [26]. Lastly, 21% of patients in the TEAMM trial withdrew consent and 44% of patients received a thalidomide-based treatment, which is not considered a standard-of-care [25]. Based on our proposed risk score, preventive strategies may be investigated in prospective clinical trials in a contemporary treatment setting. Concepts for such trials may include initial dose reduction of chemotherapy and glucocorticoids, active monitoring for patients at risk (i.e. by digital wearables), differential use of antibacterial prophylaxis, or substitution of immunoglobulins.

Another limitation is that our study could not dissect the effect of glucocorticoid dose or intensity during induction therapy, as variable glucocorticoid doses and dose intensities were used within and among the trials analyzed. These cannot be disentangled from the overall trial effect, which is accounted for in our analysis. Yet, our risk score was robust, after validation across a variety of induction regimens and accompanying glucocorticoid therapies. However, as shown previously [27, 28], treatment with low-dose glucocorticoids (i.e. dexamethasone once weekly) reduces early morbidity and mortality and is a standard-of-care.

In conclusion, our study highlights the importance of early severe infections and death in the era of novel agent-based therapy in patients with NDMM. Based on our risk score, patients at high risk of early, severe infections and death can be easily identified upfront, when evaluated for the latest quadruplet induction therapies including an anti-CD38 mAb.

### Supplementary information

Supplementary Information

## Data Availability

After the publication of this article, data collected for this analysis and related documents will be made available to others upon reasonably justified request, which needs to be written and addressed to the attention of the corresponding author, Elias K. Mai, at the following e-mail address: elias.mai@med.uni-heidelberg.de. The EMN, HOVON and GMMG, via the corresponding author Elias K. Mai, are responsible for evaluating and eventually accept or refuse every request to disclose data and their related documents, in compliance with the ethical approval conditions, in compliance with applicable laws and regulations, and in conformance with the agreements in place with the involved subjects, the participating institutions, and all the other parties directly or indirectly involved in the participation, conduct, development, management, and evaluation of this analysis.
