# Supplementary material for: Predictors of early morbidity and mortality in newly diagnosed multiple myeloma: data from five randomized, controlled, phase III trials in 3700 patients
Source: Leukemia. 2023 Dec 7;38(3):640–7. doi: 10.1038/s41375-023-02105-6 (PMC10912032; doi:10.1038/s41375-023-02105-6)

SUPPLEMENTARY INFORMATION

Predictors of early morbidity and mortality in newly diagnosed multiple myeloma: data from five randomized, controlled, phase III trials in 3700 patients

by E. K. Mai, et al.

SUPPLEMENTARY TABLES

Table S1. Characteristics and treatments in the trials included in the multi-cohort analysis.

	GMMG-HD4/HOVON-65	GMMG-MM5	GMMG-HD6	EMN02/HOVON-95	GMMG-HD7
**Recruitment period**	05/2005–05/2008	07/2010–11/2013	06/2015–09/2017	02/2011–04/2014	10/2018–09/2020
**Induction regimen**	**Arm B: PAD** bortezomib i.v. 1.3 mg/m^2 on days 1, 4, 8, 11 / doxorubicin i.v. 9 mg/m^2 on days 1–4 / dexamethasone p.o. 40 mg on days 1–4, 9–12, 17–20; repeated on day 29 (3x)	**Arms A1+B1: PAd** bortezomib i.v./s.c. 1.3 mg/m^2 on days 1, 4, 8, 11 / doxorubicin i.v. 9 mg/m^2 on days 1–4 / dexamethasone p.o. 20 mg on days 1–4, 9–12, 17–20; repeated on day 29 (3x)<br><br>**Arms A2+B2: VCD** bortezomib i.v./s.c. 1.3 mg/m^2 on days 1, 4, 8, 11 / cyclophosphamide i.v. 900 mg/m^2 on day 1 / dexamethasone p.o. 40 mg on days 1–2, 4–5, 8–9, 11–12, repeated on day 22 (3x)	**Arms A1+A2: RVd** lenalidomide p.o. 25 mg on days 1–14 / bortezomib s.c. 1.3 mg/m^2 on days 1, 4, 8, 11 / dexamethasone 20 mg on days 1–2, 4–5, 8–9, 11–12; repeat on day 22 (4x)<br><br>**Arms B1+B2: Elotuzumab-RVd** elotuzumab i.v. 10 mg/kg on days 1, 8, 15 (cycles 1+2) and on days 1 and 11 (cycles 3+4) / lenalidomide p.o. 25 mg on days 1–14 / bortezomib s.c. 1.3 mg/m^2 on days 1, 4, 8, 11 / dexamethasone 20 mg on days 1–2, 4–5, 8–9, 11–12 and 15 (only cycles 1+2); repeat on day 22 (4x)	**VCD:** bortezomib i.v. 1.3 mg/m^2 on days 1, 4, 8, 11 / cyclophosphamide i.v. 500 mg/m^2 on days 1 and 8 / dexamethasone p.o. 40 mg on days 1–2, 4–5, 8–9, 11–12; repeated on day 22 (3x *or* 4x)	**Arm IA: RVd** lenalidomide p.o. 25 mg on days 1–14 and 22–35 / bortezomib s.c. 1.3 mg/m^2 on days 1, 4, 8, 11, 22, 25, 29, 32 / dexamethasone 20 mg on days 1–2, 4–5, 8–9, 11–12, 15, 22–23, 25–26, 29–30, 32–33; repeat on day 43 (3x)<br><br>**Arm IB: Isatuximab-RVd** isatuximab i.v. 10 mg/kg on days 1, 8, 15, 22, 29 (cycle 1) and on days 1, 15, 29 (cycles 2+3) / lenalidomide p.o. 25 mg on days 1–14 and 22–35 / bortezomib s.c. 1.3 mg/m^2 on days 1, 4, 8, 11, 22, 25, 29, 32 / dexamethasone 20 mg on days 1–2, 4–5, 8–9, 11–12, 15, 22–23, 25–26, 29–30, 32–33; repeat on day 43 (3x)

**Anti-bacterial prophylaxis[a]**	Recommended	Mandatory	Mandatory	Recommended	Recommended
***N* of patients per induction treatment**	192/218 (PAD only)	596 (PAd: 296; VCD: 300)	545 (RVd: 272; elotuzumab-RVd: 273)	1491 (VCD only)	658 (RVd: 328; isatuximab-RVd: 330)
**CTCAE version**	3.0	4.0	5.0	4.0	5.0
**Trial registry**	EudraCT No. 2004-000944-26	EudraCT No. 2010-019173-16	NCT 02495922	NCT 01208766	NCT 03617731

CTCAE National Cancer Institute Common Terminology Criteria for Adverse Events, *EMN* European Myeloma Network, *GMMG* German-speaking Myeloma Multicenter Group, *HOVON* Dutch–Belgian Cooperative Trial Group for Hematology Oncology Foundation.
[a]Recommendations according to study protocols – HD4/HO-65: cotrimoxazol; MM5: cotrimoxazol or ciprofloxacin; HD6: cotrimoxazol or ciprofloxacin; EMN02/HO-95: not specified/according to local protocols; HD7: cotrimoxazol.

Table S2. Patient demographics and baseline characteristics in the trials included in the training cohort.

***n* (%)**		**GMMG-HD4**	**GMMG-MM5**	**GMMG-HD6**	**All**
**Evaluable**	Yes	192 (100)	596 (100)	545 (100)	1333 (100)
**Completed IT**	Yes	180 (93.8)	567 (95.1)	514 (94.3)	1261 (94.6)
**Median age**	Years (range)	55 (31–65)	58 (32–70)	58 (32–70)	58 (31-70)
**Age > 60 years**	No	140 (72.9)	347 (58.2)	312 (57.2)	799 (59.9)
	Yes	52 (27.1)	249 (41.8)	233 (42.8)	534 (40.1)
	Missing	-	-	-	-
**Sex**	Female	121 (63.0)	348 (58.4)	309 (56.7)	778 (58.4)
	Male	71 (37.0)	248 (41.6)	236 (43.3)	555 (41.6)
	Missing	-	-	-	-
**WHO performance status**	0–1	177 (92.2)	533 (90.5)	496 (91.0)	1206 (91.0)
	> 1	15 (7.8)	56 (9.5)	49 (9.0)	120 (9.0)
	Missing	-	7	-	7
**Body mass index**	≤ 30 kg/m^2	162 (84.4)	488 (81.9)	438 (81.0)	1088 (81.9)
	> 30 kg/m^2	30 (15.6)	108 (18.1)	103 (19.0)	241 (18.1)
	Missing	-	-	4	4
**White blood cell count**	≥ 4.0/nL	169 (88.0)	487 (81.7)	436 (80.0)	1092 (81.9)
	< 4.0/nL	23 (12.0)	109 (18.3)	109 (20.0)	241 (18.1)
	Missing	-	-	-	-
**Hemoglobin**	≥ 10.0 g/dL	121 (63.0)	394 (66.1)	385 (70.6)	900 (67.5)
	< 10.0 g/dL	71 (37.0)	202 (33.9)	160 (29.4)	433 (32.5)
	Missing	-	-	-	-
**Platelets**	≥ 150/nL	172 (89.6)	520 (87.2)	489 (89.7)	1181 (88.6)
	< 150/nL	20 (10.4)	76 (12.8)	56 (10.3)	152 (11.4)
	Missing	-	-	-	-
**Creatinine**	≤ 2.0 mg/dL	173 (90.1)	530 (88.9)	512 (93.9)	1215 (91.2)
	> 2.0 mg/dL	19 (9.9)	66 (11.1)	33 (6.1)	118 (8.8)
	Missing	-	-	-	-
**Calcium**	≤ 2.75 mmol/L	176 (92.6)	554 (93.0)	525 (96.3)	1255 (94.3)
	> 2.75 mmol/L	14 (7.4)	42 (7.0)	20 (3.7)	76 (5.7)
	Missing	2	-	-	2
**C-reactive protein**	≤ 5.0 mg/L	120 (65.2)	340 (57.7)	351 (65.0)	811 (61.8)

	> 5.0 mg/L	64 (34.8)	249 (42.3)	189 (35.0)	502 (38.2)
	Missing	8	7	5	20
**Lactate dehydrogenase**	Normal	151 (83.4)	489 (82.3)	451 (83.2)	1091 (82.8)
	> ULN	30 (16.6)	105 (17.7)	91 (16.8)	226 (17.2)
	Missing	11	2	3	16
**International Staging System**	I/II	141 (78.3)	433 (72.7)	423 (77.6)	997 (75.5)
	III	39 (21.7)	163 (27.4)	122 (22.4)	324 (24.5)
	Missing	12	-	-	12
**Cytogenetics**	Standard risk	126 (75.5)	387 (73.3)	322 (72.0)	835 (73.1)
	High risk	41 (24.6)	141 (26.7)	125 (28.0)	307 (26.9)
	Missing	25	68	98	191
**Anti-bacterial prophylaxis**	No	21 (35.6)	67 (11.2)	9 (1.6)	97 (8.1)
	Yes	38 (64.4)	529 (88.8)	536 (98.3)	1103 (91.9)
	Missing	133	-	-	133

GMMG German-speaking Myeloma Multicenter Group, *IT* induction therapy, *ULN* upper limit of normal, *WHO* World Health Organization.

Table S3. Study endpoints in the training cohort.

***n* (%)**	**GMMG-HD4**	**GMMG-MM5**	**GMMG-HD6**	**All**
Evaluable	192 (100)	596 (100)	545 (100)	1333 (100)
Severe infection (grade ≥ 3)	52 (27.1)	60 (10.1)	46 (8.4)	158 (11.8)
Death	4 (2.1)	8 (1.3)	12 (2.2)	24 (1.8)
Severe infection/death	53 (27.6)	64 (10.7)	50 (9.2)	167 (12.5)
Cause of death	(*n* = 4)	(*n* = 8)	(*n* = 12)	(*n* = 24)
Infection	2 (50.0)	4 (50.0)	9 (75.0)	15 (62.5)
Multiple myeloma	1 (25.0)	2 (25.0)	2 (16.7)	5 (20.8)
Thromboembolic event	-	2 (25.0)	1 (8.3)	3 (12.5)
Unknown	1 (25.0)	-	-	1 (4.2)

GMMG German-speaking Myeloma Multicenter Group.

Table S4. Odds ratio and 95% confidence interval estimates comparing risk score stages for endpoints severe infection, death, and severe infection/death in the training cohort.

	**Odds ratio**	**95% Confidence interval**	***p***
**Severe infection**			
High vs low risk	3.40	1.99–5.79	< 0.001
High vs intermediate risk	2.25	1.39–3.66	< 0.001
Intermediate vs low risk	1.51	0.91–2.50	0.06
**Death**			
High vs low risk	15.67	2.68–91.56	< 0.001
High vs intermediate risk	4.70	1.58–13.99	< 0.001
Intermediate vs low risk	3.33	0.51–21.57	0.13
**Severe infection/death**			
High vs low risk	3.63	2.15–6.13	< 0.001
High vs intermediate risk	2.34	1.46–3.76	< 0.001
Intermediate vs low risk	1.55	0.94–2.55	0.04

Comparison of risk groups for endpoints severe infection, death, or combined endpoint of severe infection/death.

Table S5. Distribution of baseline and risk factors in the validation cohorts.

n (%)		HOVON-65	EMN02/HOVON-95	GMMG-HD7
**Evaluable**	Yes	218 (100)	1491 (100)	658 (100)
**Completed IT**	Yes	197 (90.3)	1340 (89.9)	605 (92.0)
**Age > 60 years**	No	157 (72.0)	970 (65.1)	372 (56.5)
	Yes	61 (28.0)	521 (34.9)	286 (43.5)
	Missing	-	-	-
**WHO performance status**	0–1	184 (84.8)	1270 (85.2)	592 (90.2)
	> 1	33 (15.2)	221 (14.8)	62 (9.8)
	Missing	1	-	2
**Platelets**	≥ 150/nL	189 (87.1)	1263 (85.9)	592 (90.0)
	< 150/nL	28 (12.9)	208 (14.1)	66 (10.0)
	Missing	1	20	-
**International Staging System**	I/II	153 (78.9)	1163 (78.0)	516 (78.2)
	III	41 (21.1)	328 (22.0)	142 (21.6)
	Missing	24	-	-
**Anti-bacterial prophylaxis**	No	27 (25.7)	361 (24.5)	16 (2.4)
	Yes	78 (74.3)	1112 (75.5)	642 (97.6)
	Missing	113	18	-

EMN European Myeloma Network, *GMMG* German-speaking Myeloma Multicenter Group, *HOVON* Dutch–Belgian Cooperative Trial Group for Hematology Oncology Foundation, *IT* induction therapy, *WHO* World Health Organization.

Table S6. Study endpoints in the validation cohorts.

n (%)	HOVON-65	EMN02/HOVON-95	GMMG-HD7
**Evaluable**	218	1491	658
**Severe infection (grade ≥ 3)**	56 (25.7)	101 (6.8)	77 (11.7)
**Death**	13 (6.0)	25 (1.7)	12 (1.8)
**Severe infection/death**	61 (28.0)	118 (7.9)	81 (12.3)

EMN European Myeloma Network, *GMMG* German-speaking Myeloma Multicenter Group, *HOVON* Dutch–Belgian Cooperative Trial Group for Hematology Oncology Foundation.

Table S7. Odds ratio and 95% confidence interval estimates comparing risk score stages for endpoints severe infection, death, and severe infection/death in the HO65 trial.

	**Odds ratio**	**95% Confidence interval**	***p***
**Severe infection**			
High vs low risk	3.12	1.15–8.45	0.007
High vs intermediate risk	2.23	0.80–6.20	0.07
Intermediate vs low risk	1.40	0.56–3.53	0.39
**Death**			
High vs low risk	6.67	0.89–49.86	0.03
High vs intermediate risk	1.49	0.33–6.70	0.54
Intermediate vs low risk	4.47	0.64–31.38	0.07
**Severe infection/death**			
High vs low risk	3.24	1.21–8.65	0.005
High vs intermediate risk	1.96	0.72–5.31	0.11
Intermediate vs low risk	1.65	0.68–4.02	0.19

Comparison of risk groups for endpoints severe infection, death, or combined endpoint of severe infection/death.

Table S8. Odds ratio and 95% confidence interval estimates comparing risk score stages for endpoints severe infection, death, and severe infection/death in the EMN02/HO95 trial.

	**Odds ratio**	**95% Confidence interval**	***p***
**Severe infection**			
High vs low risk	2.36	1.25–4.46	0.002
High vs intermediate risk	1.48	0.83–2.66	0.11
Intermediate vs low risk	1.59	0.87–2.91	0.07
**Death**			
High vs low risk	5.11	1.05–24.97	0.02
High vs intermediate risk	1.17	0.40–3.43	0.73
Intermediate vs low risk	4.37	0.96–19.79	0.02
**Severe infection/death**			
High vs low risk	2.56	1.41–4.66	< 0.001
High vs intermediate risk	1.50	0.87–2.59	0.08
Intermediate vs low risk	1.70	0.97–3.01	0.03

Comparison of risk groups for endpoints severe infection, death, or combined endpoint of severe infection/death.

Table S9. Odds ratio and 95% confidence interval estimates comparing risk score stages for endpoints severe infection, death, and severe infection/death in the HD7 trial.

	**Odds ratio**	**95% Confidence interval**	***p***
**Severe infection**			
High vs low risk	3.38	1.62–7.04	< 0.001
High vs intermediate risk	2.47	1.25–4.90	0.002
Intermediate vs low risk	1.37	0.66–2.82	0.31
**Death**			
High vs low risk	5.08	0.71–36.52	0.05
High vs intermediate risk	2.56	0.52–12.54	0.17
Intermediate vs low risk	1.98	0.26–15.19	0.43
**Severe infection/death**			
High vs low risk	3.68	1.78–7.62	< 0.001
High vs intermediate risk	2.49	1.28–4.84	0.001
Intermediate vs low risk	1.48	0.73–3.02	0.20

Comparison of risk groups for endpoints severe infection, death, or combined endpoint of severe infection/death.

SUPPLEMENTARY FIGURE

Fig. S1. Logistic regression analyses of factors influencing risk of severe infection, death, or combined endpoint of severe infection/death during induction therapy for each trial included in the training cohort.

A Forest plots on factors associated with risk of severe infection. **B** Forest plots on factors influencing risk of death. **C** Forest plots on factors influencing risk of severe infection and/or death. Analyses are presented for each trial in the training cohort (HD4, MM5, and HD6) and overall. All logistic regression analyses including the complete training cohort accounted for trial effects. *P* values from univariable analysis were adjusted for multiple testing.

BMI body mass index, *CI* confidence interval, *ISS* International Staging System, *LDH* lactate dehydrogenase, *ULN* upper limit of normal, *WHO* World Health Organization.

Fig. 1A

Variable	Trial	*N*/Severe infections	Odds ratio (95% CI)	*p*	IA *p*
ISS stage III	HD4	180/48	1.31 (0.60–2.79)	0.951	0.481
	MM5	596/60	2.23 (1.29–3.83)	0.019	
	HD6	545/46	3.00 (1.61–5.54)	0.009	
	All	1321/154	2.17(1.51–3.12)	< 0.001	
Platelets (< 150/nL)	HD4	192/52	0.94 (0.31–2.50)	0.951	0.458
	MM5	596/60	3.23 (1.71–5.91)	0.006	
	HD6	545/46	3.24 (1.50–6.57)	0.023	
	All	1333/158	2.49 (1.59–3.83)	< 0.001	
Age (> 60 years)	HD4	192/52	1.29 (0.64–2.57)	0.951	0.659
	MM5	596/60	2.26 (1.32–3.91)	0.018	
	HD6	545/46	1.82 (1.00–3.37)	0.142	
	All	1333/58	1.83 (1.29–2.59)	0.003	
WHO performance status (> 1)	HD4	192/52	2.57 (0.89–7.32)	0.525	0.782
	MM5	589/58	1.68 (0.72–3.52)	0.313	
	HD6	545/46	2.44 (1.03–5.26)	0.142	
	All	1326/156	2.07 (1.23–3.35)	0.021	
Calcium (> 2.75 mmol/L)	HD4	190/50	3.07(1.04–9.11)	0.525	0.659
	MM5	596/60	1.64 (0.62–3.72)	0.321	
	HD6	545/46	3.10 (0.92–8.58)	0.142	
	All	1331/156	2.23 (1.22–3.90)	0.027	
Hemoglobin (< 10.0 g/dL)	HD4	192/52	1.10 (0.57–2.09)	0.951	0.659
	MM5	596/60	1.35 (0.78–2.31)	0.321	
	HD6	545/46	1.80 (0.96–3.30)	0.142	
	All	1333/158	1.38 (0.97–1.95)	0.150	
Serum creatinine (> 2.0 mg/dL)	HD4	192/52	0.75 (0.22–2.11)	0.951	0.458
	MM5	596/60	2.57 (1.28–4.90)	0.030	
	HD6	545/46	1.24 (0.33–3.47)	0.938	
	All	1333/158	1.57 (0.91–2.59)	0.183	
C-reactive protein (> 5.0 mg/L)	HD4	184/50	1.08 (0.55–2.11)	0.951	0.782
	MM5	589/59	1.36 (0.80–2.33)	0.321	
	HD6	540/46	1.49 (0.80–2.71)	0.295	
	All	1313/155	1.31 (0.93–1.86)	0.183	
LDH (> ULN)	HD4	181/50	0.97 (0.39–2.25)	0.951	0.481
	MM5	594/59	2.08 (1.12–3.75)	0.057	
	HD6	542/46	1.09 (0.47–2.28)	0.967	
	All	1317/155	1.40 (0.91–2.11)	0.183	
Sex (male)	HD4	192/52	1.10 (0.57–2.09)	0.951	0.458
	MM5	596/60	0.53 (0.29–0.93)	0.059	
	HD6	545/46	1.01 (0.55–1.85)	0.967	
	All	1333/158	0.80 (0.56–1.13)	0.278	
White blood cell count (< 4.0/nL)	HD4	192/52	1.24 (0.47–3.06)	0.951	0.659
	MM5	596/60	0.59 (0.25–1.24)	0.284	
	HD6	545/46	0.95 (0.39–2.03)	0.967	
	All	1333/158	0.81 (0.49–1.30)	0.466	
BMI (> 30 kg/m²)	HD4	192/52	1.73 (0.75–3.84)	0.839	0.458
	MM5	596/60	0.70 (0.31–1.43)	0.345	
	HD6	541/46	1.60 (0.78–3.11)	0.295	
	All	1329/158	1.19 (0.76–1.79)	0.476	
High-risk cytogenetics	HD4	167/51	0.94 (0.43–1.98)	0.951	0.458
	MM5	528/54	0.62 (0.29–1.20)	0.284	
	HD6	447/38	1.58 (0.78–3.11)	0.295	
	All	1142/143	0.94 (0.62–1.41)	0.770	

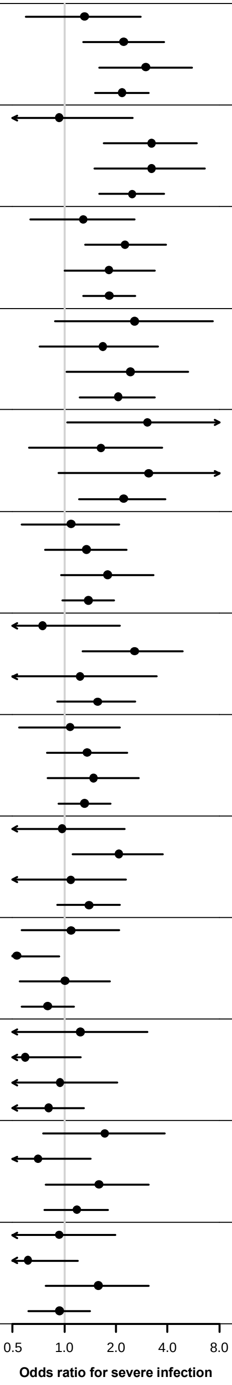

Fig. 1B

Variable	Trial	*N*/Deaths	Odds ratio (95% CI)	*p*	IA *p*
Severe infections	HD4	192/4	6.58 (1.05–69.06)	0.488	0.747
	MM5	596/8	9.42 (2.37–37.45)	0.017	
	HD6	545/12	24.31 (7.68–87.73)	< 0.001	
	All	1333/24	15.21 (6.58–36.85)	< 0.001	
WHO performance status (> 1)	HD4	192/4	5.16 (0.48–33.99)	0.488	0.963
	MM5	589/7	7.70 (1.68–32.45)	0.055	
	HD6	545/12	8.07 (2.44–25.19)	0.009	
	All	1326/23	7.10 (2.96–16.30)	< 0.001	
ISS stage III	HD4	180/4	3.72 (0.56–24.83)	0.488	0.963
	MM5	596/8	4.27 (1.12–18.59)	0.100	
	HD6	545/12	4.94 (1.62–16.04)	0.028	
	All	1321/24	4.57 (2.05–10.51)	0.001	
Severe thromboembolic events	HD4	192/4	2.10 (0.02–22.11)	0.896	0.747
	MM5	596/8	40.80 (6.42–211.91)	0.008	
	HD6	545/12	6.63 (1.22–25.16)	0.080	
	All	1333/24	7.86(2.33–21.79)	0.008	
Platelets (< 150/nL)	HD4	192/4	0.91 (0.01–9.05)	0.952	0.747
	MM5	596/8	4.46 (1.02–17.12)	0.119	
	HD6	545/12	4.86 (1.36–15.23)	0.065	
	All	1333/24	3.53 (1.39–8.18)	0.030	
Hemoglobin (< 10.0 g/dL)	HD4	192/4	0.72 (0.07–4.48)	0.919	0.747
	MM5	596/8	3.12 (0.82–13.55)	0.201	
	HD6	545/12	3.38 (1.1–10.94)	0.080	
	All	1333/24	2.53 (1.14–5.72)	0.057	
BMI (> 30 kg/m²)	HD4	192/4	2.32 (0.22–14.70)	0.712	0.747
	MM5	596/8	4.64 (1.18–18.23)	0.100	
	HD6	541/12	1.57 (0.39–5.07)	0.629	
	All	1329/24	2.36 (0.97–5.34)	0.123	
C-reactive protein (> 5.0 mg/L)	HD4	184/3	3.19 (0.41–35.38)	0.518	0.747
	MM5	589/8	0.87 (0.20–3.28)	0.903	
	HD6	540/12	2.59 (0.85–8.37)	0.198	
	All	1313/23	1.84 (0.81–4.19)	0.268	
Sex (male)	HD4	192/4	1.72 (0.26–11.36)	0.829	0.963
	MM5	596/8	2.23 (0.59–9.69)	0.401	
	HD6	545/12	1.32 (0.43–4.06)	0.629	
	all	1333/24	1.66 (0.75–3.74)	0.352	
Serum creatinine (> 2.0 mg/dL)	HD4	192/4	3.95 (0.37–25.60)	0.518	0.963
	MM5	596/8	1.60 (0.17–7.48)	0.848	
	HD6	545/12	2.01 (0.21–8.86)	0.629	
	All	1333/24	1.79 (0.47–5.05)	0.500	
Calcium (> 2.75 mmol/L)	HD4	190/4	5.51 (0.51–36.51)	0.488	0.747
	MM5	596/8	0.76 (0.01–6.25)	0.903	
	HD6	545/12	3.44 (0.36–15.65)	0.385	
	All	1331/24	1.95 (0.39–6.24)	0.500	
LDH (> ULN)	HD4	181/3	0.70 (0.01–7.44)	0.927	0.747
	MM5	594/8	0.92 (0.10–4.29)	0.929	
	HD6	542/12	2.68 (0.76–8.31)	0.221	
	All	1317/23	1.45 (0.50–3.57)	0.562	
High-risk cytogenetics	HD4	167/3	5.30 (0.68–59.13)	0.488	0.747
	MM5	528/6	0.74 (0.08–3.76)	0.903	
	HD6	447/9	1.39 (0.33–4.99)	0.629	
	All	1142/18	1.41 (0.51–3.57)	0.562	
Age (> 60 years)	HD4	192/4	2.74 (0.41–18.19)	0.518	0.747
	MM5	596/8	2.21 (0.58–9.62)	0.401	
	HD6	545/12	0.70 (0.20–2.15)	0.629	
	All	1333/24	1.30 (0.57–2.89)	0.563	
White blood cell count (< 4.0/nL)	HD4	192/4	0.78 (0.01–7.71)	0.929	0.923
	MM5	596/8	1.72 (0.31–6.84)	0.732	
	HD6	545/12	0.65 (0.07–2.77)	0.629	
	All	1333/24	0.83 (0.22–2.29)	0.737	

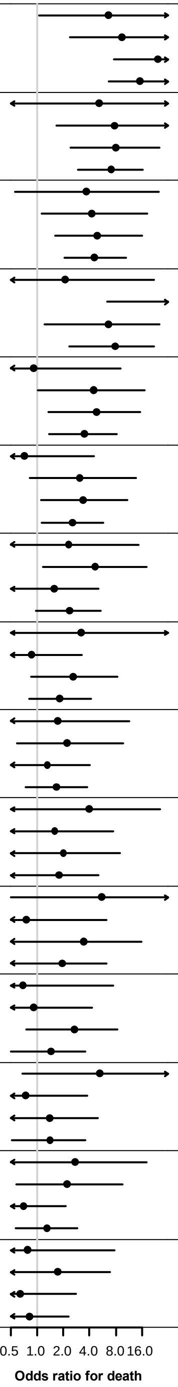

Fig. 1C

Variable	Trial	*N*/Events	Odds ratio (95% CI)	*p*	IA *p*
ISS stage III	HD4	180/49	1.27 (0.57–2.69)	0.951	0.581
	MM5	596/64	2.46 (1.45–4.16)	0.006	
	HD6	545/50	3.11 (1.70–5.62)	0.004	
	All	1321/163	2.30 (1.61–3.26)	< 0.001	
Platelets (< 150/nL)	HD4	192/53	0.91 (0.30–2.42)	0.951	0.581
	MM5	596/64	3.23 (1.73–5.83)	0.004	
	HD6	545/50	3.29 (1.57–6.55)	0.014	
	All	1333/167	2.52 (1.63–3.84)	< 0.001	
WHO performance status (> 1)	HD4	192/53	2.50 (0.86–7.10)	0.585	0.714
	MM5	589/61	1.83 (0.82–3.75)	0.223	
	HD6	545/50	3.00 (1.35–6.19)	0.035	
	All	1326/164	2.31 (1.41–3.68)	0.004	
Age (> 60 years)	HD4	192/53	1.25 (0.62–2.47)	0.951	0.670
	MM5	596/64	2.21 (1.31–3.76)	0.012	
	HD6	545/50	1.64 (0.92–2.95)	0.202	
	All	1333/167	1.74 (1.24–2.44)	0.004	
Calcium (> 2.75 mmol/L)	HD4	190/51	2.98 (1.01–8.83)	0.585	0.684
	MM5	596/64	1.51 (0.58–3.42)	0.406	
	HD6	545/50	2.81 (0.84–7.74)	0.202	
	All	1331/165	2.08 (1.14–3.62)	0.047	
Hemoglobin (< 10.0 g/dL)	HD4	192/53	1.05 (0.54–2.00)	0.951	0.670
	MM5	596/64	1.49 (0.88–2.52)	0.223	
	HD6	545/50	1.86 (1.02–3.34)	0.137	
	All	1333/167	1.45 (1.03–2.03)	0.070	
Serum creatinine (> 2.0 mg/dL)	HD4	192/53	0.73 (0.22–2.05)	0.951	0.581
	MM5	596/64	2.63 (1.33–4.94)	0.020	
	HD6	545/50	1.12 (0.30–3.13)	0.905	
	All	1333/167	1.56 (0.92–2.56)	0.169	
LDH (> ULN)	HD4	181/50	0.97 (0.39–2.25)	0.951	0.670
	MM5	594/63	1.89 (1.02–3.37)	0.115	
	HD6	542/50	1.31 (0.61–2.60)	0.614	
	All	1317/163	1.42 (0.93–2.12)	0.169	
C-reactive protein (> 5.0 mg/L)	HD4	184/50	1.08 (0.55–2.11)	0.951	0.828
	MM5	589/63	1.37 (0.81–2.30)	0.281	
	HD6	540/50	1.39 (0.77–2.50)	0.390	
	All	1313/163	1.29 (0.92–1.81)	0.197	
BMI (> 30 kg/m²)	HD4	192/53	1.67 (0.73–3.71)	0.951	0.670
	MM5	596/64	0.96 (0.47–1.82)	0.905	
	HD6	541/50	1.60 (0.80–3.03)	0.291	
	All	1329/167	1.30 (0.86–1.94)	0.272	
White blood cell count (< 4.0/nL)	HD4	192/53	1.21 (0.45–2.97)	0.951	0.684
	MM5	596/64	0.64 (0.28–1.29)	0.281	
	HD6	545/50	0.85 (0.35–1.81)	0.819	
	All	1333/167	0.80 (0.48–1.26)	0.404	
Sex (male)	HD4	192/53	1.05 (0.54–2.00)	0.951	0.670
	MM5	596/64	0.66 (0.38–1.13)	0.223	
	HD6	545/50	1.04 (0.57–1.85)	0.905	
	All	1333/167	0.87 (0.62–1.21)	0.442	
High-risk cytogenetics	HD4	167/51	0.94 (0.43–1.98)	0.951	0.581
	MM5	528/56	0.66 (0.32–1.26)	0.281	
	HD6	447/40	1.64 (0.82–3.16)	0.290	
	All	1142/147	0.98(0.65–1.45)	0.918	

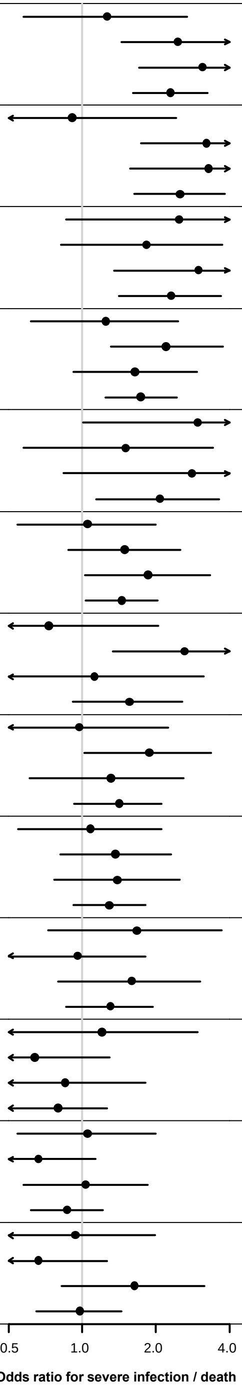

Supplement: Supplementary file 1 — Supplementary Information [file 41375_2023_2105_MOESM1_ESM.pdf]
